# Technological Advances in Intra-Operative Navigation: Integrating Fluorescence, Extended Reality, and Artificial Intelligence

**DOI:** 10.3390/jcm14238574

**Published:** 2025-12-03

**Authors:** Edward Murphy, Ronan A. Cahill

**Affiliations:** 1 UCD Centre for Precision Surgery, 47 Eccles Street, Phibsboro, D07 Y9AW Dublin, Ireland; 2 Department of Surgery, Mater Misericordiae University Hospital, D07 R2WY Dublin, Ireland

**Keywords:** surgical navigation, fluorescence-guided surgery, augmented reality, artificial intelligence, indocyanine green

## Abstract

Surgical navigation is a rapidly advancing area of innovation that has extended from its roots as a tool based on rigid anatomical landmarks into the complex domain of soft-tissue surgery. Three complementary technologies—fluorescence-guided surgery (FGS), extended reality (XR), and artificial intelligence (AI)—are converging to provide real-time visualisation and decision support. FGS, most often currently using indocyanine green (ICG), is now widely applied for perfusion assessment, lymphatic mapping, and biliary anatomy delineation, with growing evidence of improved surgical safety and efficacy. Targeted fluorophores are under development to enable disease-specific imaging, while XR platforms can overlay three-dimensional reconstructions onto the operative field to enhance spatial orientation. AI offers the potential to standardise interpretation, reduce variability, and analyse complex intra-operative datasets to guide surgical decisions. Despite these advances, significant barriers remain before broad clinical deployment, including technical limitations, limited high-quality evidence, training demands and regulatory and ethical challenges. The near future of surgical navigation lies in integrating FGS, XR, and AI into a cohesive system that enhances precision, safety, and outcomes and remains adaptable to future imaging and therapeutic innovations.

## 1. Introduction

Surgical navigation is a rapidly evolving field of innovation within modern healthcare. Broadly, the Oxford English Dictionary defines navigation as “*the process of accurately ascertaining one’s position and planning and following a route*”. This principle, in theory, is as applicable to the operating theatre as it is to maritime or other areas of geographical exploration—every manoeuvre in surgery depends on knowing exactly where you are in relation to your intended path and your critical surroundings. A global positioning system (GPS) in frequent use in daily life displays such information, including positioning along the intended journey with details of the current location, and it can update in real time to adapt to new information. Surgeons planning their intraoperative route can now also equip themselves similarly, with real-time personalised information to support and document their intraoperative path and decision-making.

Historically, surgical navigation found its first foothold in specialties such as orthopaedic, neurosurgery and otorhinolaryngological surgery [1], where bony structures provide fixed, easily registered landmarks for image guidance. By contrast, general and abdominal surgery present a more complex challenge: soft tissues are mobile, deformable and subject to constant physiological motion from respiration, peristalsis, and surgical manipulation. This has generally limited the translation of rigid-body navigation systems into the soft-tissue domain, although pioneering work in liver and adrenal surgery has demonstrated real-time navigation capability some years ago [2,3]. Additionally, over the past three decades, general surgery has shifted from predominantly comprising open approaches, rich in tactile feedback, to minimally invasive surgery (MIS) accesses, where haptic cues are reduced, and now robotic platforms have moved the surgeon even further from direct tissue contact. Digital visualisation has become commonplace, and so augmentation and real-time navigation have become increasingly possible and potentially critical as compensatory and evolutionary adjuncts. Advances in high-resolution laparoscopic and robotic imaging, optical fluorescence techniques, and computational image processing are creating new possibilities to deploy digital guidance technologies at scale.

Specifically, a new generation of technologies—fluorescence-guided surgery (FGS), extended reality (XR), and artificial intelligence (AI)—are now converging to transform intra-operative navigation. FGS, typically using indocyanine green (ICG), provides both static and dynamic visualisation of anatomy (including concealed structures) and physiology (including arterial, venous and lymphatic flow) with targeted fluorophores offering the potential for disease-specific visualisation. Three-dimensional reconstruction methods can provide digital and physical models to allow surgeons to plan and rehearse operative strategies [4,5,6]. AR can superimpose such preoperative imaging and segmented anatomy models onto the live operative field [7], while AI is beginning to interpret complex intra-operative datasets, converting raw visual information into actionable surgical guidance. This article examines how these modalities are developing, how they may be integrated, and what barriers remain before they can be routinely deployed. It is important to realise, as you read about these technologies, that they are not occurring in their own vacuum, and each individual technology described below is being developed alongside the others, working together to provide synergy.

## 2. Current State of Surgical Navigation

**Figure 1:**
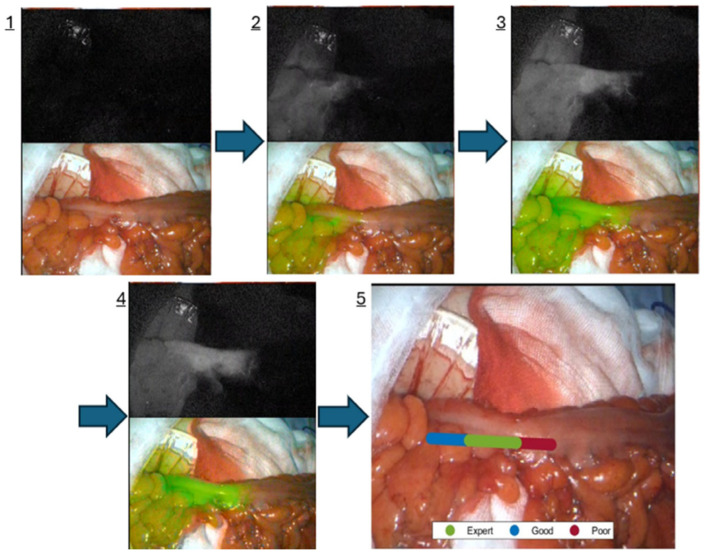
FGS showing bowel perfusion prior to transection (image (**1**–**4**), NIR in black and white, mask overlay in colour image), image (**5**) shows an AI-recommended transection point. McEntee et al., Colorectal Diseases, April 2025 [15].

FGS has emerged as the most clinically mature technology in this space. ICG, a water-soluble near-infrared dye first approved for hepatic function testing in the 1950s, has become the workhorse modality and will likely continue to be important even while other targeted fluorophores emerge from clinical studies. Administered intravenously, it binds to plasma albumin and so remains intravascular until cleared by the liver and emits near-infrared light when excited at ~805 nm. This allows surgeons to visualise blood flow and tissue perfusion as well as biliary structures because of its excretory pathways in real time using near-infrared camera systems integrated into laparoscopic and robotic platforms. Similarly, after interstitial injection, lymphatic processes, including lymph node concentration, can be identified, and ureters can be rendered fluorescent when the agent is directly instilled cystoscopically. Equipped with laparoscopic and robotic near—infrared cameras available via most commercial companies, surgeons can visualise real-time perfusion, lymphatics and anatomical planes that remain invisible under white light. Its broad applications are increasingly being standard of care including for perfusion assessment checking during colorectal anastomoses (with reduced postoperative leak rates in randomised controlled trials) (Figure 1) [8,9,10], lymphatic mapping in gastrointestinal malignancies (for better oncological staging) [11], biliary anatomy delineation during cholecystectomy (for better critical view of safety delineation) [12,13] and ureteric identification in complex pelvic dissections [14].

FGS’s usefulness will also be aided by tailored molecular/cellular targeted fluoro-phores. Recent examples include OTL38, which binds to folate receptors overexpressed in certain cancers, and IRDye 800CW, which can be conjugated to tumour-specific antibodies [16], and SGM-101, which is a fluorochrome-labelled anti-carcinoembryonic antigen (CEA) monoclonal antibody. These agents have shown promise for real-time tumour deposit detection (for which OTL38 has regulatory approval in ovarian and lung cancer) [17] and also improved lymph node identification in early-phase clinical trials. SGM-101 is currently recruiting an international phase III trial assessing curative resection in primary, recurrent and metastatic colorectal malignancy.

For these new dyes and for anatomy identification with ICG (such as in biliary and lymphatic mapping), FGS is used as a static qualitative inference, with the dye being given hours, if not days, pre-operatively. For perfusion, FGS is real-time, intraoperative guidance. When viewed through this prism, FGS acts as a gateway digital technology through which additional computational methods can be applied, potentially enabling soft tissue surgery “catch up” with neurosurgical, orthopaedic and otolaryngological procedures in terms of navigational assistance. In this way, too of the role of ICG, as notably the most available dye, can evolve too. Where it was often thought previously that ICG is overly limited by its lack of specificity for pathological characterisation when it is given preoperatively, recent studies have shown that intraoperative ICG perfusion analysis with AI can be utilised to identify specific structural alterations in tissues, such as the disorganised neo-vasculature and capillary permeability that hallmark malignant transformation. Exploitation of these structural abnormalities can be used to differentiate malignant tissue from its benign counterpart in a manner analogous to dynamic MRI imaging but directly usable during operations and endoscopic evaluations [18]. It is not an infallible technology; we must still account for maximum fluorescence intensity [19], fluorescence diffusion [20] and background auto-fluorescence, but this is a promising new advance in FGS that can expand in the coming years. In particular, ratiometric sensors (using dual wavelength analysis) and dyes that are excited in the NIR II region (1000–1700 nm wavelength, further from visible wavelength) may offer an imaging advantage [21,22].

XR is an umbrella term that encompasses the new technological field of computer-generated simulation, including virtual reality (VR), augmented reality (AR) and mixed reality (MR). VR, like that seen in computer gaming, is an immersive simulated world. Crucially, it may mimic the real world but cannot interact with it. AR and MR are similar in that they both provide a computer-generated world superimposed on our real world to enhance perception. The simulated interface in this case is based on the real-world image and can interact or overlay onto real-world structures/anatomy. The point of difference between AR and MR is that MR allows the two worlds to interact with each other. Most notably for this discussion, MR allows the surgeon to interact with the superimposed simulated reality.

VR, therefore, is likely more applicable to surgical education rather than true surgical navigation. Harnessing its gaming background can create immersive operative scenarios and experiences that have been shown to improve surgical training and performance in both technical skills and procedural confidence [23,24]. Gamification will be the next step advancement for VR in surgical training, like Laptitude (Grendel Games BV, Leeuwarden, The Netherlands). VR gamification has already shown improvements in knowledge and skill acquisition [25] and some systems have already proven construct validity [26].

**Figure 2:**
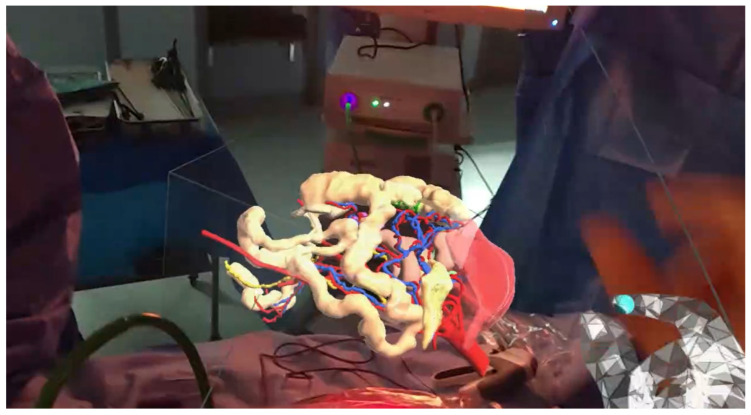
Vascular anatomy during laparoscopic right hemicolectomy. Three-dimensional reconstruction by VisiblePatient (Visible Patient Lab, Strasbourg, France), displayed through Microsoft HoloLens 2 headset (Microsoft, Redmond, WA, USA). A Moynihan et al., Colorectal Diseases, October 2023 [31].

AR and MR’s benefit for surgery is that their superimposition of extra layers of information on the real world enhances the surgeon’s understanding of the operative scene. This may be beneficial for operative planning, intraoperative decision-making and MDT planning thanks to recent improvements in 3D anatomical imaging. Three-dimensional rendering and printing saw a technological explosion in the mid-2000s [27]; now, its primary application is to create personalised patient-specific anatomical 3D models from radiological DICOM images [28,29]. Although physical 3D models and prostheses have clinical relevance across many specialities, they have been predominantly applied to date in those surgical specialities dealing with bony/rigid anatomy. In general surgery, 3D printing’s influence has been less evident so far (with evidence that surgeons prefer interacting with digital 3D models more than physically printed models [30]), but it is developing, including in surgical training and operative planning of anatomical complexities, such as planning for complex colon (Figure 2), liver or pancreas resections [27,31].

Three-dimensional anatomical analysis has been harnessed by devices such as Holocare Studio (Holocare AS, Oslo, Norway) in liver and biliary surgery, providing MR environments that can help guide intraoperative dissection while preserving critical structures in real-time via an interface that can superimpose vascular segment maps and tumour boundaries directly onto the operative scene as viewed through a headset [32]. Importantly, too accurate anatomical imaging can predict physiological patterns such as perfusion, where high correlation between preoperative vascular maps and intraoperative FGS sufficiency analysis has been shown [6].

AI is becoming increasingly influential across the surgical continuum, with applications ranging from pre-operative planning and intra-operative assessment to surgical training and education. While its reach is broad, its most disruptive potential may lie within surgical navigation. In this context, AI promises to transform intra-operative visualisation by providing consistent, real-time interpretation of complex anatomical and physiological signals. Unlike human observation, which is inherently variable and subject to fatigue, AI models apply standardised analytical frameworks across every case. This does not make AI adversarial to surgical judgement, but rather complementary and does not replace the expertise of the surgeon. Early integration of AI into FGS shows great potential in enhancing perfusion analysis [33], and its synergy with AR/MR platforms may be critical for communication of this information in real time through the combination of these modalities into a unified intra-operative “navigation stack”.

The foundation of AI in surgical navigation is computer vision. One of the earliest and most validated applications is instrument identification and tracking, which provides the basis for operative assistance devices and, ultimately, automated robotic functions [34,35,36]. Platforms such as the Senhance Robotic System (Asensus Surgical, Durham, NC, USA) and the Moon Surgical Maestro system replicate the role of a laparoscopic assistant through AI-driven tracking of instrument movement, demonstrating how machine vision can already be applied to operative workflow.

This is quite a basic use of such technology; the next frontier for AI in navigation is environmental understanding: analysing tissue characteristics, perfusion patterns, and anatomical planes to deliver context-aware decision support. Systems such as the AR-based Eureka (Anaut Inc., Tokyo, Japan) platform have demonstrated improved intra-operative visualisation of ureters and pelvic autonomic nerves [37,38]. Although long-term outcome data are awaited, recent studies suggest that enhanced nerve preservation correlates with improved functional recovery in pelvic surgery [39], supporting the expectation that AI-augmented overlays will provide tangible benefit in colorectal and urological operations.

Achieving the required level of precision is technically challenging. Soft tissues deform, shift with respiration, and change configuration under retraction, meaning that overlays generated from pre-operative imaging often lose alignment intra-operatively. Overcoming this requires advanced registration algorithms and significant computational power. AI-driven solutions are beginning to address this problem: for example, Laplante et al. developed and validated GoNoGoNet, a decision-support tool for laparoscopic cholecystectomy that predicts safe and unsafe dissection zones with greater than 90% accuracy [40]. Such systems exemplify how AI can move from passive annotation toward active safety guidance. Excitingly, too, recent breakthrough advancements demonstrate that fully autonomous operative steps can be practically achieved via the application of AR and robotic surgical systems, although the regulatory approval of such systems may be fraught [41].

It is at the interface of edge computing and real-time intra-operative analysis that perhaps the greatest near-term opportunity lies. The ability of AI to interpret anatomical cues or process fluorescence signals and deliver actionable decision support directly to the surgeon—via AR overlays or intra-operative masking—marks a paradigm shift in surgical navigation. Within our own department, we are currently conducting two multicentre trials that integrate all three technologies: indocyanine green (ICG) fluorescence for tissue perfusion assessment, AI algorithms for perfusion classification with decision support, and AR platforms for intra-operative visualisation. Such multidisciplinary integration illustrates the future direction of surgical navigation, where the surgeon’s judgement is enhanced—not replaced—by an intelligent, context-sensitive guidance system.

## 3. Barriers to Adoption

To this point, we have discussed the considerable promise these new technologies hold, but there are several barriers that must be addressed before FGS, AR and AI can be widely adopted in surgical practice.

FGS seems the closest to widespread deployment, with the now robust evidence base that exists for its use as a perfusion assessment adjunct in restorative rectal surgery [8,9,42] and lymphatic mapping in gynaecological malignancy, and indeed many commercial surgical imaging systems include this technological capability (although often as an optional extra rather than as standard). However, even if available in a department, often use is limited to selected individuals, suggesting still issues with awareness of the benefit or learning curve concerns. Approval and access to ICG have broadly improved over recent years, at least in the USA and Europe, although the lack of availability and cost can still be barriers to full utilisation as well as workflow considerations. XR and AI use have been less successful in breaking into the general surgery ecosystem.

Technical limitations for the emerging computational technologies, however, remain significant.

In FGS, fluorescence intensity can be influenced by the camera angle, working distance, and ambient light, making quantitative analysis challenging. Maximum fluorescence intensity can be negated by using relative perfusion parameters, using a “normal” area of tissue for relative comparison or viewing the fluorescence as a proportion of change over time. FGS imaging can also be limited by phenomena of photobleaching and quenching, where fluorescence intensity is degraded by light or molecule interaction, respectively. These have to be accounted for but will likely be improved with the development of new dye agents. NIR I (700–950 nm wavelength) dyes are particularly vulnerable to low signal to background contrast, poor tissue penetration and high autofluorescence, which may be limited, for example, by the introduction of NIR II dyes [43].

AI models require vast, well-annotated datasets to train effectively and performance can be inconsistent when applied to different imaging systems or patient populations. This poses a number of concerns, first is the data collection, it is imperative that there is a strict data management plan in place as software usage can be significantly affected by organisational resources such as training, staffing, workflow and processes [44,45].

Once the data collected is appropriate, then the next challenge is how it is processed, including annotation. Reproducibility of results is crucial but can be limited by model drift, whereby there is degradation of the machine learning model due to changes in data or input variables [46]. This can be difficult to monitor for but it is crucial that there is a strong governance framework with emphasis on regular performance assessment and retraining with new data inputs [46,47].

Many models rely on unsupervised deep learning or so-called “black box” intelligence [48], where decision pathways are opaque even to developers—let alone to surgeons or patients. This lack of transparency risks undermining trust in AI guidance [49]. Explainable AI may improve clarity in this regard, but it has its own inherent risks. So-called “white box” AI, using simpler decision trees or linear regression models, can be explained but has far less predictive power [50]. Whereas to make “black box” AI explainable, we have to use post hoc analysis of global explanations (feature importance or rule extraction) or local explanations (Local Interpretable Model-agnostic Explanations [51] or Shapley Additive Explanations [52]).

Similarly, XR and AR overlays are prone to registration drift as tissues move, deform, or swell intra-operatively.

Evidence gaps persist. For AR, the literature remains dominated by feasibility and pilot studies with limited evidence of routine clinical deployment and effective improvements in such parameters as operative time, blood loss or complication rates with its real-time applications. Devices like Eureka and GoNoGoNet have shown promising results in anatomical annotation and guidance, but these tools remain untested in controlled trials capable of proving patient benefit.

Training demands are also considerable. Surgeons will need to learn how to interpret new types of intra-operative information, manage multiple visual inputs without distraction, and adapt their decision-making processes accordingly [53]. A fundamental question remains: at what point might decision support become intrusive, distracting or even unsafe? This threshold may vary by procedure, by surgeon or by the individual’s familiarity with digital technologies. Yet surgical curricula currently contain little structured training in the interpretation or safe use of these systems.

Ethical and legal barriers may prove the most complex. Questions of liability loom large. Healthcare practitioners are trained to take responsibility for their decisions, but should this change with AI based decision supports. If an AI-based decision support system contributes to an adverse outcome, responsibility could fall on the surgeon, the developer, the vendor, the healthcare institution, or regulators. No consensus currently exists [53], but ideally they will share liability risks [54].

Regulatory frameworks are only beginning to adapt and may shift again when the European Union AI Act comes into effect for AI medical devices in August 2026 [55]. Some navigation devices have already achieved regulatory approval, though transparency varies. In Europe, CE-mark approval lists are difficult to access, while in the United States, the Food and Drug Administration (FDA) maintains a public database. As of July 2025, 1247 AI-enabled medical devices had FDA clearance—956 of which were in radiology, but only 6 in general surgery [56]. This imbalance highlights both the rapid expansion of AI in imaging and the relative immaturity of adoption within operative navigation.

## 4. Discussion

The trajectory of surgical navigation is clearly shifting from isolated innovation to integrated multimodal platforms. FGS has matured and demonstrates real clinical utility in perfusion assessment and anatomical delineation, and with the advent of targeted fluorophores, continues to close the specificity gap in oncologic applications. XR platforms now extend these visual cues into immersive, spatially accurate environments, improving intra-operative orientation and preoperative planning. Meanwhile, AI is emerging as the interpretive layer—processing intra-operative data streams, standardising analysis, and transforming complex imaging into actionable decision support.

Despite this convergence, translation to clinical practice remains inconsistent. There is a clear discordance between recognition of digital innovation and its practical adoption in surgery. The Royal College of Surgeons in Ireland recently reported that 97% of respondents acknowledged AI’s future importance, yet over two-thirds had no exposure, and nearly three-quarters felt their specialty was underinvesting [57].

While enthusiasm for innovation is high, adoption lags due to technical, structural, and cultural barriers. Training deficits, interoperability challenges, and a lack of validated clinical endpoints continue to slow implementation. The persistent divide between recognition of technological value and its integration into operative workflows underscores the need for structured digital education, standardised datasets, and cross-disciplinary collaboration. Without proactive investment in these areas, the transformative potential of FGS, XR, and AI risks remaining unrealised.

What is most striking is that these technologies are no longer developing in isolation. AI analysis of ICG fluorescence, communicated through AR interfaces, demonstrates how the “navigation stack” is evolving into a collaborative ecosystem. This synergy promises not only incremental improvement but also a step-change in surgical practice, particularly in soft-tissue surgery where tactile feedback has become more limited. However, the future of widespread adoption will require evidence of improved patient outcomes through well-designed trials, as well as transparency in algorithmic decision-making and regulatory harmonisation. The development of explainable, adaptive AI—capable of continuous learning under governance frameworks—will be central to this progress.

## 5. Conclusions

Surgical navigation is advancing toward an era of intelligent integration, where fluorescence, extended reality, and AI converge within a unified “navigation stack” to deliver real-time, context-aware guidance. Building and validating this stack—supported by transparency, training, and collaboration—will be essential to transform digital tools into trusted partners in precision surgery.

## Abbreviations

The following abbreviations are used in this manuscript:

MIS	Minimally invasive surgery
AI	Artificial intelligence
XR	Extended reality
VR	Virtual reality
AR	Augmented reality
MR	Mixed reality
FGS	Fluorescence-guided surgery
ICG	Indocyanine green
GPS	Global positioning system
